# Systematic Review with Meta-Analysis: Efficacy and Safety of Direct-Acting Antivirals for Chronic Hepatitis C Genotypes 5 and 6

**DOI:** 10.1155/2019/2301291

**Published:** 2019-11-11

**Authors:** Ong The Due, Usa Chaikledkaew, Anne Julienne M. Genuino, Abhasnee Sobhonslidsuk, Ammarin Thakkinstian

**Affiliations:** ^1^Mahidol University Health Technology Assessment (MUHTA) Graduate Program, Bangkok, Thailand; ^2^Health Strategy and Policy Institute, Vietnam Ministry of Health, Hanoi, Vietnam; ^3^Social and Administrative Pharmacy Excellence Research (SAPER) Unit, Department of Pharmacy, Faculty of Pharmacy, Mahidol University, Bangkok, Thailand; ^4^Health Technology Assessment Unit, Health Regulation Team, Department of Health Philippines, Manila, Philippines; ^5^Division of Gastroenterology and Hepatology, Department of Medicine, Faculty of Medicine Ramathibodi Hospital, Mahidol University, Bangkok, Thailand; ^6^Section for Clinical Epidemiology and Biostatistics, Faculty of Medicine Ramathibodi Hospital, Mahidol University, Bangkok, Thailand

## Abstract

Direct-acting antivirals (DAAs) are modern treatments for chronic hepatitis C infection, but majority of available evidence on its treatment effect covers genotypes 1 to 4. Therefore, the efficacy and safety of DAAs for genotypes 5 and 6 need to be analysed. Studies were identified from Medline, Scopus, and CENTRAL and a Chinese database CNKI, from inception until Dec 4, 2018. Clinical trials were included if they enrolled patients with genotypes 5 and/or 6 infection, any type of second-generation DAAs was studied, and sustained virological response was assessed at the 12^th^ week after treatment (SVR12) as outcome measure. Meta-analysis using *metaprop* statistical program was applied for pooling proportions if data were sufficient (i.e., at least 2 studies). Thirteen studies were included in the analysis. Four studies assessed the efficacy of four DAA regimens in genotype 5 patients, which were mainly sofosbuvir (SOF) plus pegylated-interferon/ribavirin (PR) or other DAAs, with SVR12 ranging from 94.4% to 100%. Twelve studies assessed the efficacy of seven DAA regimens among genotype 6 patients, but only two DAA regimens (i.e., SOF + PR and SOF/ledipasvir) had sufficient data for pooling. The pooled SVR12 rates (95% CI) were 99.6% (92.2 to 100) for SOF + PR and 99.2% (96.5 to 100) for SOF/ledipasvir. No treatment-related serious adverse event was reported, while the nonserious adverse events were comparable to other genotypes. In conclusion, DAAs are effective and may be safe for the treatment of chronic hepatitis C genotypes 5 and 6. However, our evidence is based on noncomparative studies; hence, further larger-scale randomized controlled trials in these genotypes are still required.

## 1. Introduction

Hepatitis C virus (HCV) is classified into six major genotypes based on sequence diversities [1–3]. Genotype 1 is the most common infection globally (46%), whereas the prevalence of other genotypes varies by geographical distributions, i.e., genotype 2 (13%) in West Africa; genotype 3 (22%) in South Asia and parts of Scandinavia; genotype 4 (13%) in Central and North Africa [4]; and genotype 5 (1%) and 6 (2%) in South Africa and Southeast Asia [4]. The available evidence on HCV treatment efficacy mainly focuses on patients with genotype 1 infection because genotypes 5 and 6 are less common in developed countries [5].

The initial and conventional treatment for HCV was the combination therapy of pegylated-interferon (PegIFN) and ribavirin (RBV). While this regimen has been shown to achieve sustained virological response (SVR) rates from 40% to 65%, it was poorly tolerated and was associated with severe adverse events [6]. Improved chronic HCV treatment has been seen with the introduction of direct-acting antiviral drugs (DAAs) or oral medicines directly targeting the HCV genome [6]. Compared to interferon-based regimens, DAAs increased SVR rates with shorter treatment duration and fewer side-effects [7]. As of August 2017, thirteen DAAs had been approved by the Food and Drug Administration (FDA) for HCV treatments which were categorized into four classes: (a) NS3/4A class, i.e., simeprevir (SMV), paritaprevir (PTV), grazoprevir (GZR), voxilaprevir (VOX), and glecaprevir (GCV); (b) NS5A class, i.e., ledipasvir (LDV), ombitasvir (OBV), daclatasvir (DCV), elbasvir (EBR), velpatasvir (VEL), and pibrentasvir (PBV); (c) NS5B nucleoside class, i.e., sofosbuvir (SOF); and (d) NS5B non-nucleoside class, i.e., dasabuvir (DSV). These DAAs could be combined in duplet or triplet regimens to achieve higher SVR rates up to 95% [7, 8].

Although there have been clinical trials evaluating the efficacy of DAAs in HCV infections with genotypes 5 and 6, to the best of our knowledge, there has been no systematic review and meta-analysis on this topic. In addition, treatment guidelines show that the treatments for both genotypes 5 and 6 are identical [6, 9–11]. With this, we aimed to synthesize all available clinical trials to estimate the efficacy (i.e., SVR rate) and safety of available DAA-based treatment regimens for chronic HCV with genotypes 5 and 6 infections. The results of this study would be useful in informing clinical practice for HCV genotypes 5 and 6 treatment, future economic evaluations, and clinical trials on HCV treatment.

## 2. Materials and Methods

The study was conducted in accordance with the Preferred Reporting Items for Systematic Reviews and Meta-Analyses (PRISMA) guidelines [12]. The review protocol was registered at PROSPERO International Prospective Register of systematic reviews, and their protocol was followed in conducting this review (Registration number: CRD42017074273).

### 2.1. Identification of Studies

A systematic search in Medline (via PubMed), Scopus, and the Cochrane Central Register of Controlled Trials (CENTRAL) databases was performed with last search on December 4, 2018, to identify relevant studies. Search terms were constructed based on (a) patients—HCV, hepacivirus, and hepatitis C; (b) interventions—including both generic names, i.e. sofosbuvir, ledipasvir, daclatasvir, and velpatasvir, and brand names, i.e., Solvadi, Harvoni, Daklinza, and Epclusa; and (c) study design—clinical trial, including both randomized trial and nonrandomized trial. No search filters were applied.

Because genotypes 5 and 6 are relatively restricted in geographical exposure, we also attempted to explore regional and country databases where these genotypes exist. As China is known to rank second in the world's total publications [13], we searched the China National Knowledge Infrastructure (CNKI)—one of the largest databases in China recommended for non-Chinese-speaking researchers [14, 15]. However, our search was limited because the database did not allow desired advanced search; therefore, only general keywords such as “hepatitis C” and “genotype” were used. No search filters were further applied. The details of the search strategies for these databases are described in [Supplementary-material supplementary-material-1].

### 2.2. Selection of Studies

Two authors (OTD and AJG) independently selected studies by screening the titles and abstracts based on eligibility criteria. Full articles were retrieved if decision could not be made based on abstracts. Any disagreements were resolved by consensus with a third reviewer (AT). Clinical trials were included if they met all of the following criteria: (a) patients with chronic HCV infection genotypes 5 and/or 6 with or without cirrhosis, including treatment-naïve or treatment-experienced; (b) patients received one or more than one type of second-generation DAAs, and (c) patients reported SVR12 rate for genotype 5 or 6. Studies were excluded if they involved patients coinfected with other viruses (e.g., HIV and HBV) or had advanced diseases (e.g., liver or kidney transplantation and hemodialysis), or if they reported in non-English language (see [Supplementary-material supplementary-material-1] for full inclusion/exclusion criteria).

### 2.3. Interventions

Interventions of interest were the second-generation DAAs because the first-generation DAAs (i.e., boceprevir and telaprevir) are no longer used for HCV treatment [6, 16]. The second-generation DAAs currently recommended by international guidelines for treatment of HCV genotypes 5 and 6 include SOF, LDV, DCV, and VEL [6, 9–11]. Treatment regimens of interest were single (SOF + RBV ± PegIFN), duplet (i.e., SOF/LDV ± RBV, SOF/VEL ± RBV, SOF + DCV ± RBV), or triplet DAAs (i.e., SOF/VEL/VOX).

### 2.4. Outcome of Interest

The primary outcome of interest was SVR at the 12^th^ week after treatment (SVR12) which was originally defined by individual studies as HCV RNA level lower than detectable level (i.e., 15 or 25 IU/mL). The secondary outcomes of interest were nonsevere adverse events (AE) and serious adverse events (SAE). Nonsevere AEs included headache, upper respiratory tract infection, fatigue, nausea, or insomnia, while SAEs were abdominal pain, hemorrhagic shock, agitation, or urinary tract infection.

### 2.5. Data Extraction

Two authors (OTD and AJG) independently extracted information from all selected studies using a predesigned data extraction form ([Supplementary-material supplementary-material-1]). The following data were extracted: general information, study design, participant characteristics (e.g., mean age, percent of male, and percent of ethnicity), baseline clinical data (e.g., HCV RNA, treatment history, and percent of cirrhosis), details of the intervention and comparator, and outcome measures. Study authors were contacted for any unclear or missing information. Further disagreements were resolved by consensus with a third reviewer (AT).

### 2.6. Risk of Bias Assessment

The validity of each trial was independently assessed by two authors (OTD and AJG). Any disagreements were resolved by consensus with a third reviewer (AT).

For randomized trials, the revised Cochrane risk-of-bias tool for randomized trials (RoB 2) was used [17] which evaluated on the following domains: (a) bias arising from the randomization process; (b) bias due to deviations from intended interventions; (c) bias due to missing outcome data; (d) bias in measurement of the outcome; and (e) bias in selection of the reported result. For each domain, an outcome of “low risk,” “high risk,” or “some concerns” was recorded and overall assessment of risk of bias was then determined for each study.

For nonrandomized trials, the Cochrane risk of bias tool for nonrandomized studies (ROBINS-I) was used [18] which assessed on the following domains: (a) bias due to confounding; (b) bias in selection of participants into the study; (c) bias in classification of interventions; (d) bias due to deviations from intended intervention; (e) bias due to missing data; (f) bias in measurement of outcomes; and (g) bias in selection of the reported result. For each domain, an outcome of low, moderate, serious, critical or no information for risk of bias was recorded. An overall risk of bias was then determined per study through combination of the seven domains.

### 2.7. Statistical Analysis

Meta-analysis was performed separately by HCV genotypes (i.e., genotypes 5 and 6) if data were available and sufficient for pooling. The rates of SVR12, AEs, and SAEs were estimated and pooled across studies through the command *metaprop*, which is a statistical program developed to perform meta-analysis of proportions in STATA [19]. The fixed-effect model using inverse variance method was applied if there was no heterogeneity. Otherwise, the random-effects model using Der-Simonian and Laird was used. Heterogeneity of treatment effects was assessed using Cochrane *Q* test and *I*^2^ statistics where heterogeneity was considered present if the *Q* test was significant (*P* < 0.10). Sources of heterogeneity were explored by fitting covariables—mean age, percent of male, treatment history (i.e., naïve and experienced), percent of cirrhosis, and baseline HCV RNA log_10_ and percent of patients with HCV RNA ≥ 800,000—through meta-regression if data were available. A covariable was considered as a source of heterogeneity if the regression coefficient was significant or Tau^2^ decreased more than 50% after inclusion in the meta-regression model. Pooling within subgroups of covariable should be able to reduce the degree of heterogeneity (*I*^2^) within subgroups.

Publication bias was assessed using funnel plot and Egger's test. If asymmetry was present from either a funnel plot or Egger's test, a contour-enhanced funnel plot was used to explore whether the asymmetry was due to publication bias or heterogeneity.

All analyses were performed using STATA version 14.0. *P* values < 0.05 were considered statistically significant for all analyses unless otherwise stated.

## 3. Results

**Figure 1:**
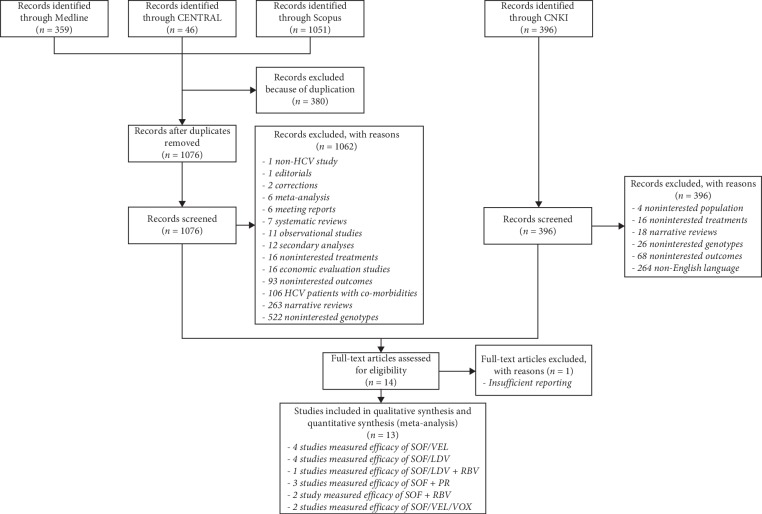
Flow of study selections.

A total of 1456 studies from Medline, CENTRAL, and Scopus plus 396 additional studies from the Chinese database (CNKI) were identified (Figure 1), but only 13 studies finally met our inclusion criteria and were used for further analysis [20–32].

The baseline characteristics of 13 included studies are shown in Table 1. Only one study [20] and three studies [24, 30, 32] designed a separate treatment arm for genotype 5 and genotype 6, respectively. In the other nine studies [21–23, 25–29, 31], these genotypes were mixed with genotypes 1 to 4 because patients with genotypes 5 and 6 were relatively few due to low prevalence. Most of these studies reported baseline characteristics of patients as an aggregate number of all genotypes in the treatment arm, which caused difficulties in extracting specific data for genotypes 5 and 6. Only three authors [21, 28, 31] responded when disaggregated data were requested, hence the baseline characteristics of several studies were still missing. Overall, majority of patients were older than 50 years, treatment-naïve, noncirrhotic, and with high viral load.

None of the 13 studies measured the comparative efficacy and safety between treatment arms, but the SVR12 and the AE/SAE rates of individual arms were reported instead. To make use of these data, we pooled SVR12 and AE/SAE rates of each regimen using data from individual treatment arms. Furthermore, it is noteworthy that in case a study containing arms of the same regimen but different durations [25, 28, 30, 31], only one arm applying common durations was used for pooling with other studies.

### 3.1. Risk of Bias Assessment

Among the 13 included studies, three [22, 23, 28] were randomized trials and 10 [20, 21, 24–27, 29–32] were nonrandomized trials.

Among the three randomized trials, only Everson et al. [22] reported randomization and allocation process [22], thus having low risk of bias for randomization process; while the other two studies [23, 28] were considered having some concerns to determine their risk of bias. In the domain pertaining to the deviations from intended interventions, all three studies were assessed as low risk of bias because no deviations occurred. For the three remaining domains, all three studies were considered to have low risk of bias because there were no missing data, the measurement of SVR12 was defined clearly, and all prespecified outcomes were reported. Consequently, only the study by Everson et al. [22] had an overall low risk of bias, while the two other studies were still considered having some concerns because the risk of bias in the domain of randomization process cannot be determined (Table 2).

Of the 10 nonrandomized trials, six studies [20, 21, 24, 26, 27, 29] did not have comparative arms; therefore, it was not feasible to assess for risk of bias in confounding domain. Hence, although they were graded as low risk of bias in six remaining domains, their overall biases could not be concluded. The four remaining nonrandomized trials [25, 30–32] contained comparative arms; therefore, we were able to assess bias due to confounding. All of them were assessed with serious risk of bias in this domain due to the existence of prognostic factors that determined which regimen a patient would receive such as cirrhosis status [25, 30, 31], antiviral treatment history [30, 31], contraindications for RBV or PegIFN [30, 31], or comorbidities [31]. Although they had low risk of bias in the six remaining domains, their overall biases were graded as serious risk of bias (Table 3). Plausible reason for this result might be that all studies did not aim to assess the comparative efficacy and safety between different regimens, but intended to measure the efficacy and safety of a specific regimen. As such, only SVR12 and AE/SAE rates, instead of a comparative ratio (e.g., risk ratio and odds ratio), were measured and reported.

### 3.2. Efficacy of DAA Regimens on HCV Genotypes 5 and 6 Patients

#### 3.2.1. HCV Genotype 5

Four studies assessed the efficacy of four DAA regimens on genotype 5 patients. Each regimen had only one study; therefore, there were insufficient data for pooling SVR12 for any regimen (Table 4).

#### 3.2.2. HCV Genotype 6

Twelve studies assessed the efficacy of seven DAA regimens on genotype 6 patients. Two DAA regimens, i.e., SOF + PR and SOF/LDV, were feasible for pooling (Table 4).

**Figure 2:**
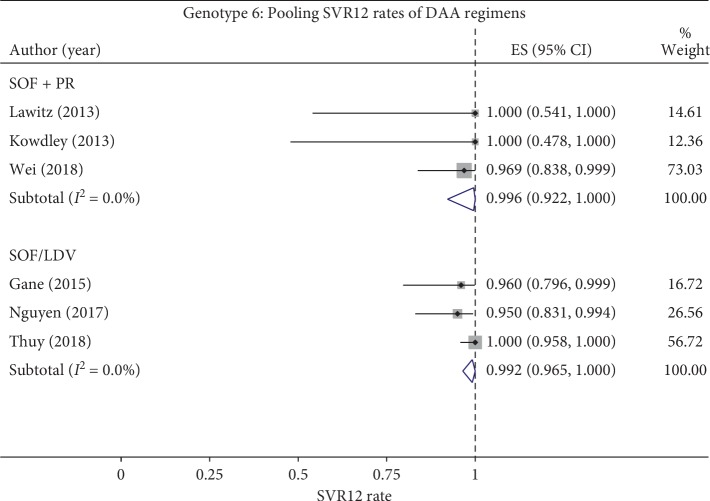
Pooling SVR12 rates of DAA regimens on HCV genotype 6. SVR12, sustained virological response rates at 12 weeks; HCV, hepatitis C virus; DAA, direct-acting antiviral; SOF, sofosbuvir; LDV, ledipasvir; PR, PegIFN + ribavirin.

*(1) SOF* *+* *PR*. The SVR12 rates were pooled from three studies (*n* = 43, Table 4 and Figure 2) without heterogeneity (*I*^2^ = 0%), which resulted in a pooled SVR12 rate of 99.6% (95% CI, 92.2 to 100).

*(2) SOF/LDV*. As mentioned above, in case a study containing arms of the same regimen but different durations, only one arm applying the common duration was used for pooling with other studies. Therefore, only the 12-week arms of Nguyen et al. [30] and Thuy et al. [31] were selected to be pooled with the 12-week arm of Gane et al. [24]. Consequently, the SVR12 rates of 12-week SOF/LDV regimen were pooled (*n* = 151, Table 4 and Figure 2) without heterogeneity (*I*^2^ = 0%), which resulted in a pooled SVR12 rate of 99.2% (95% CI, 96.5 to 100).

Furthermore, we performed additional analyses for genotypes 5 and 6 (Appendix [Supplementary-material supplementary-material-1], Appendix Figures [Supplementary-material supplementary-material-1] and [Supplementary-material supplementary-material-1]), which pooled the SVR12 rates by groups of DAA regimens (i.e., singlet, doublet, and triplet DAA regimens). Consequently, in genotype 5, only doublet regimen was feasible for pooling which resulted in a pooled SVR12 rate of 96.1% (95% CI, 90.0 to 99.7). In genotype 6, the pooled SVR12 rates for single, duplet, and triplet DAA regimens were 100% (95% CI, 95.1 to 100), 100% (95% CI, 100 to 100), and 100% (95% CI, 100 to 100), respectively.

### 3.3. Safety of DAA Regimens on HCV Genotypes 5 and 6 Patients

#### 3.3.1. HCV Genotype 5

Only one study by Abergel et al. [20] reported the safety of DAA regimen—SOF/LDV (Table 5)—in terms of both AE and SAE. Among 41 patients receiving treatment, 33 patients (80%) were reported to have at least an AE. The most common of which were asthenia in 16 patients (39%), headache in 11 patients (27%), and fatigue in four patients (10%). Only one patient (2.4%) was reported to have an SAE which is depression. Abergel et al., however, stated that this was considered not related to SOF/LDV regimen [20].

Regarding other DAA regimens, although we were not able to extract the AE/SAE rate specifically among genotype 5, the average AE/SAE rate on mixed genotypes can be retrieved. Of these, the overall AE rates of SOF + PR, SOF/VEL, and SOF/VEL/VOX regimens were 95%, 78%, and 72%, respectively, while the corresponding SAE rates were 1%, 2%, and 3%, respectively. The most common AEs included fatigue, headache, nausea, insomnia, diarrhea, or upper respiratory tract infection [23, 26, 29].

#### 3.3.2. HCV Genotype 6

Six studies reported the safety profile of five DAA regimens on genotype 6 patients (Table 5). The pooling for the SAE rates was feasible only for the SOF/LDV regimen. Further, it is the only regimen with reported AE rates for genotype 6.

*(1) SOF/LDV*. The rates of AE were reported in two studies [24, 30] with the most common AEs being fatigue, upper respiratory tract infection, diarrhea, headache, insomnia, and nausea. All of them were graded as mild to moderate in severity. Meanwhile, the rates of SAEs were reported by all three studies. However, the SAEs reported by Nguyen et al. [30] (i.e., one leg fracture due to a mechanical fall and one bleeding gastric ulcer related to helicobacter pylori infection) were considered not related to SOF/LDV regimen. Similarly, the SAEs reported by Gane et al. [24], which included hemorrhagic shock, hemorrhoidal hemorrhage, and urinary tract infection, were also judged not related to SOF/LDV regimen.

**Figure 3:**
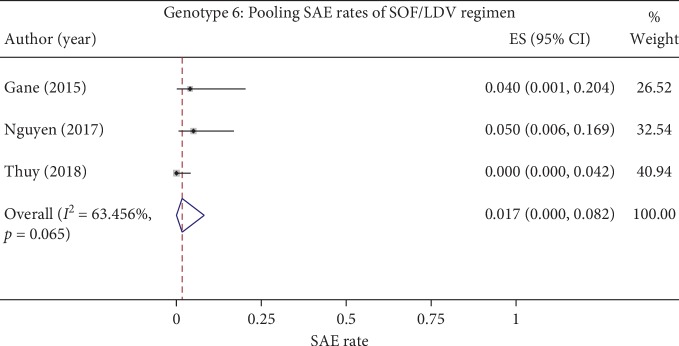
Pooling SAE rates of SOFL/LDV regimen on HCV genotype 6. SAE, serious adverse event; HCV, hepatitis C virus; SOF, sofosbuvir; LDV, ledipasvir.

The SAE rates of the 12-week SOF/LDV regimen were pooled from three studies (*n* = 151, Table 5 and Figure 3). The I^2^ was 63.5%, and the *P* value of the Cochrane *Q* test was lower than 0.1, which indicated heterogeneity. Therefore, the random-effects model was applied for pooling which resulted in a pooled SAE rate of 1.7% (95% CI, 0 to 8.2). In order to explore the source of heterogeneity, a sensitivity analysis was conducted where the study by Thuy et al. [31] was excluded from pooling. As a result, the pooled SAE rate was 4.5% (95% CI, 0.4 to 11.6) without heterogeneity (I^2^ = 0%) (Appendix [Supplementary-material supplementary-material-1]), indicating that the study by Thuy et al. [31] might be the source of heterogeneity.

Furthermore, for regimens whose specific AE rates on genotype 6 cannot be retrieved, the average AE rates on mixed genotypes were extracted. The results were SOF + RBV, 46% to 80% [28, 32]; SOF + PR, 94% to 99% [27, 29, 32]; SOF/VEL, 69% to 81% [21–23, 26]; and SOF/VEL/VOX, 67% to 80% [25, 26]. The most common AEs were similar among regimens, which include fatigue, headache, nausea, insomnia, and diarrhea, and these were more common in regimens with RBV and/or PegIFN, or regimens with longer treatment duration. However, all AEs were considered mild to moderate severity in all studies and did not require treatment discontinuation or dose modification.

Lastly, we also performed additional analyses for genotype 6 (Appendix [Supplementary-material supplementary-material-1], Appendix [Supplementary-material supplementary-material-1]) which pooled the SVR12 rates by groups of DAA regimens (i.e., singlet, doublet, and triplet DAA regimens). Only doublet regimen was feasible for pooling which resulted in a pooled SVR12 of 0.0% (95% CI, 0.0 to 0.0).

### 3.4. Publication Bias

**Figure 4:**
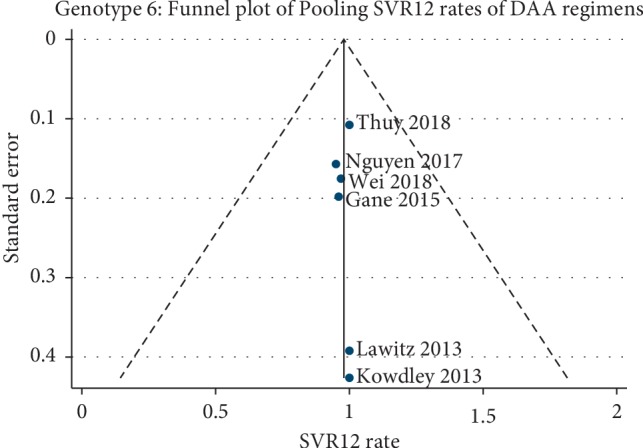
Funnel plot of pooling SVR12 of DAA regimens on HCV genotype 6. SVR12, sustained virological response rates at 12 weeks; HCV, hepatitis C virus; DAA, direct-acting antiviral.

The funnel plot of pooling SVR12 rates of SOF + PR regimen (*N* = 3 studies) and SOF/LDV regimen (*N* = 3 studies) on genotype 6 was constructed (Figure 4). The plot was symmetrical and showed no small study effect for the pooling. In addition, Egger's test was assessed which also suggested no evidence of asymmetry (*P* value = 0.741). However, the funnel plot was not constructed for pooling SAE rates of SOF/LDV regimen, due to the very small volume of studies (*N* = 3 studies).

In addition, the funnel plots for the additional analyses were also constructed (Appendix Figures [Supplementary-material supplementary-material-1]–[Supplementary-material supplementary-material-1]) which all indicated symmetry and showed no small study effect. Egger's tests also suggested no evidence of asymmetry in all funnel plots.

## 4. Discussion

We performed a systematic review and meta-analysis of efficacy and safety of DAA regimens on genotypes 5 and 6 patients by pooling SVR12 and AE/SAE rates wherever feasible. In terms of efficacy, our results indicated high efficacy of DAA regimens (i.e., SOF + PR, SOF/LDV, and SOF/VEL ± VOX) on genotype 5 patients, with the minimum SVR12 rate of 94.4%. Likewise, for genotype 6, all DAA regimens (i.e. SOF + RBV, SOF + PR, SOF/LDV ± RBV, and SOF/VEL ± VOX) showed high efficacy, where SVR12 rates ranged from 95% to 100%. Due to the small number of studies in each regimen, only two regimens SOF + PR and SOF/LDV were pooled, which resulted in SVR12 rates of 99.2% (95% CI, 96.5 to 100) and 99.6% (95% CI, 92.2 to 100), respectively. The above results suggest that DAA regimens are efficacious for both genotypes 5 and 6 patients, with slightly higher efficacy reported for genotype 6.

In terms of safety, our results indicated that DAA regimens were safe on genotype 5 and 6 patients, with an SAE rate of 0% in all regimens except SOF/LDV whose pooled SAE rate was 1.7% (95% CI, 0 to 8.2). However, all SAEs reported with SOF/LDV were considered not related to the treatment [20, 25, 30]; therefore, it might not affect the safety profile of the regimen. Regarding the AE rates, only SOF/LDV regimen had data for genotypes 5 and 6 specifically, while for other regimens, the average AE rates were extracted instead. Although all the AE rates were mostly higher than 50% and could be as high as 99%, they were considered mild to moderate severity in all studies and did not require treatment discontinuation or dose modification. The most common AEs were similar among regimens which included fatigue, headache, nausea, insomnia, and diarrhea and occurred at higher rate in regimens with RBV, PegIFN, or regimens with longer duration.

In comparison with other genotypes, DAA regimens tended to result in higher efficacy on genotypes 5 and 6. First, as regards SOF + RBV regimen, the meta-analysis by Bayatpoor et al. [33] reported pooled SVR12 rates on genotypes 2 and 3 at 92% and 55–81% (this depended on treatment duration), respectively, which were lower compared to our analysis for genotype 6 at 100% SVR12 rate. In addition, the meta-analysis by Morisco et al. [34] also reported a lower pooled SVR12 rate on genotype 3 at 79%.

With regard to SOF + PR regimen, the meta-analysis by Dolatimehr et al. [35] reported a pooled SVR12 rate of 88.5% on genotype 1, Bayatpoor et al. [33] at 95% on genotype 2, and two other meta-analyses reported 92.5% and 93% on genotype 3 [33, 36]. Meanwhile, our results indicated SVR12 rates ranging from 96.9% to 100% on genotypes 5 and 6, which were higher than genotypes 1, 2, and 3.

Moreover, regarding the efficacy of SOF/LDV ± RBV regimen, the meta-analysis by Morisco et al. [34] reported pooled SVR12 rate at 83.7% for SOF/LDV on genotype 3. In addition, several meta-analyses [37–41] reported pooled SVR12 rates ranging from 95–99.7% of SOF/LDV ± RBV on genotype 1. In our study, the SVR12 rates of SOF/LDV ± RBV ranged from 95–100% on genotypes 5 and 6 which were comparable with genotype 1, but higher than genotype 3.

In terms of the SOF/VEL regimen, the meta-analysis by Ahmed et al. [42] on genotypes 1 to 4 reported pooled SVR12 rates at 98.2%, 99.4%, 94.7%, and 99.6%, respectively. In our study, the SVR12 rates on genotypes 5 and 6 were 97.1% and 100% which were comparable with genotypes 1, 2, and 4, but slightly higher than genotype 3.

Our results have demonstrated that DAA regimens are considered effective and safe for both genotypes 5 and 6 patients, which supports the current recommendations of international guidelines on using DAA regimens on these genotypes [6, 9–11]. Furthermore, the efficacy and safety of single, doublet, or triplet DAA regimens are not much different; therefore, policy makers, especially in resource-limited settings, could seek the most affordable DAA regimens in treatment for genotypes 5 and 6, without compromising their efficacy and safety. Lastly, these results can provide clinical evidence for future economic evaluations of DAA regimens, especially in countries with high prevalence of genotypes 5 and 6 HCV patients.

## 5. Strengths and Limitations of the Study

To the best of our knowledge, this is the first systematic review and meta-analysis of the treatment efficacy and safety of DAA regimens for genotypes 5 and 6 patients. We performed a comprehensive search on three international databases and one local database to identify relevant studies which included all recommended DAA regimens for genotypes 5 and 6 patients. Clinically important outcomes were considered, which included both efficacy (SVR) and safety (AE and/or SAE). We also attempted to pool SVR12 and AE rates.

There are several limitations in this study that need to be addressed. First, we pooled SVR12 and AE rates using data from individual treatment arms only. Relative treatment effects for both efficacy and safety were not estimated and compared directly, with the reason that there was lack of available comparative studies. Secondly, the number of patients with genotypes 5 and 6 are relatively small (ranging from 1 to 86 patients in a treatment arm) because most of the included studies originally focused on patient groups of mixed genotypes. As a result, in some cases, an insufficient number of studies were available for pooling SVR12/AE/SEA rates and for constructing funnel plots. Third, because the characteristics of patients with genotypes 5 and 6 were mostly not reported, we were not able to pool SVR12 and AE rates by patients' treatment history (treatment-naive vs. treatment-experienced), cirrhosis status (cirrhosis vs. noncirrhosis), or other important patient characteristics. Fourth, in this study, we only focused on HCV patients with no comorbidities. In general, genotypes 5 and 6 are still under-researched; therefore, studies focusing on their subpopulation (i.e., HCV with comorbidities) might be rare. Fifth, our study did not use mortality or development of HCV-related complications as a primary outcome, instead we considered SVR12, which is a surrogate outcome, because we obtained data from all trials of DAA regimens which were mostly conducted in a short-term period (normally around 24 weeks) [43]. Therefore, there were no data on long-term outcomes. However, existing studies indicate that SVR12 is associated with reduced mortality and reduced risk of progression to HCV-related complications and therefore using a surrogate outcome would be justifiable [44, 45]. Lastly, we excluded articles in Chinese language because we did not have a Chinese language reviewer in our team, which was mentioned as one of the core requirements for extracting data from Chinese articles [15]. Options such as Google translate were found to be unreliable in translating Chinese language because it often resulted in inaccurate data [46]. However, recent evidence suggests that restricting a meta-analysis to English studies does not introduce bias to its results [47].

## 6. Conclusion

Our systematic review and meta-analysis indicated that DAA regimens are effective and safe as first-line treatment for chronic HCV infection in genotypes 5 and 6. The SVR12 rates of DAA regimens on genotypes 5 and 6 are found to be at least similar or higher than other genotypes and do not vary much among the different regimens. No treatment-related serious adverse event was reported, while the rates of nonserious adverse events were comparable to other genotypes. Our results would be useful in clinical practice especially in resource-limited settings where policy makers may opt to consider the most affordable drugs in treatment for genotypes 5 and 6 without compromising their efficacy and safety. Further, this systematic review and meta-analysis has provided evidence to support recommendations of international guidelines on these genotypes. However, our evidence is based on noncomparative studies; therefore, further larger-scale randomized controlled trials in genotypes 5 and 6 alongside economic evaluations are required.

## Figures and Tables

**Table 1:** Baseline characteristics of the included studies.

Study	Regimen^*∗*^	Duration (weeks)	Genotype	*n*	Age (mean)	Male (%)	Prior HCV treatment (%)		Cirrhosis (%)	Viral load	
							Naïve	Experienced		HCV RNA log_10_ IU/mL (mean)	HCV RNA ≥ 800,000 IU/mL (%)
Lawitz et al. [29]	SOF + PR	12	5	1	—	—	100	0	—	—	—
	SOF + PR	12	6	6	—	—	100	0	—	—	—
Kowdley et al. [27]	SOF + PR	24	6	5	—	—	100	0	0	-	—
Gane et al. [24]	SOF/LDV	12	6	25	51	64	92	8	8	6.7	—
Curry et al. [21]	SOF/VEL	24	6	1	67	100	100	0	100	6.5	100
Everson et al. [22]	SOF/VEL	12	6	5	54	80	100	0	0	6.7	80
	SOF/VEL	12	6	4	57	100	25	75	100		
	25 mg^*∗∗*^									6.1	75
Feld et al. [23]	SOF/VEL	12	5	35	—	—	—	—	—	—	—
	SOF/VEL	12	6	41	—	—	—	—	—	—	—
Gane et al. [25]	SOF/VEL/VOX	8	6	1	—	—	100	0	100	—	—
	SOF/VEL/VOX	12	6	2	—	—	0	100	50	—	—
Abergel et al. [20]	SOF/LDV	12	5	41	—	51	51	49	22	—	—
Lai et al. [28]	SOF + RBV	12	6	3	—	—	100	0	—	—	—
	SOF + RBV	16	6	4	—	—	100	0	—	—	—
	SOF + RBV	24	6	4	—	—	100	0	—	—	—
Jacobson et al. [26]	SOF/VEL/VOX	8	5	18	—	—	—	—	—	—	—
	SOF/VEL/VOX	8	6	30	—	—	—	—	—	—	—
	SOF/VEL	12	6	9	—	—	—	—	—	—	—
Nguyen et al. [30]	SOF/LDV	8	6	20	57	55	100	0	0	6.2	—
	SOF/LDV	12	6	40	59	60	62	38	68	6.6	—
Wei et al. [32]	SOF + PR	12	6	32	38	47	69	31	6	6.5	84
	SOF + RBV	24	6	4	36	75	50	50	0	6.4	50
Thuy et al. [31]	SOF/LDV	12	6	86	52	47	92	8	2	6.7	63
	SOF/LDV + RBV	12	6	39	59	39	33	67	56	6.7	85
	SOF/LDV	24	6	20	63	30	65	35	90	6.5	70
	SOF/LDV + RBV	24	6	30	64	40	70	30	97	6.7	82

SOF, sofosbuvir; VEL, velpatasvir; LDV, ledipasvir; VOX, voxilaprevir; PR, PegIFN + ribavirin; RBV, ribavirin. ^*∗*^Standard dose of each drug is as follows: SOF, 400 mg per day; VEL, 100 mg per day; LDV, 90 mg per day; VOX, 100 mg per day; PegIFN 180 *μ*g per week; RBV 1000–1200 mg per day. ^*∗∗*^Standard dose of VEL is 100 mg per day. In this study (Everson 2005), a VEL dose of 25 mg per day was experimented.

**Table 2:** Risk of bias assessment for individual studies (randomized trials).

Study	Bias arising from the randomization process	Bias due to deviations from intended interventions	Bias due to missing outcome data	Bias in measurement of the outcome	Bias in selection of the reported result	Overall bias
Everson et al. [22]	Low risk	Low risk	Low risk	Low risk	Low risk	Low risk
Feld et al. [23]	*Some concerns*	Low risk	Low risk	Low risk	Low risk	Some concerns
Lai et al. [28]	*Some concerns*	Low risk	Low risk	Low risk	Low risk	Some concerns

**Table 3:** Risk of bias assessment for individual studies (nonrandomized trials).

Study	Bias due to confounding	Bias in participant selection	Bias in classification of interventions	Bias due to departures from intended interventions	Bias due to missing data	Bias in measurement of outcomes	Bias in selection of the reported result	Overall bias
Lawitz et al. [29]	NA	Low risk	Low risk	Low risk	Low risk	Low risk	Low risk	NA
Kowdley et al. [27]	NA	Low risk	Low risk	Low risk	Low risk	Low risk	Low risk	NA
Gane et al. [24]	NA	Low risk	Low risk	Low risk	Low risk	Low risk	Low risk	NA
Curry et al. [21]	NA	Low risk	Low risk	Low risk	Low risk	Low risk	Low risk	NA
Abergel et al. [20]	NA	Low risk	Low risk	Low risk	Low risk	Low risk	Low risk	NA
Jacobson et al. [26]	NA	Low risk	Low risk	Low risk	Low risk	Low risk	Low risk	NA
Gane et al. [25]	Serious risk	Low risk	Low risk	Low risk	Low risk	Low risk	Low risk	Serious risk
Nguyen et al. [30]	Serious risk	Low risk	Low risk	Low risk	Low risk	Low risk	Low risk	Serious risk
Wei et al. [32]	Serious risk	Low risk	Low risk	Low risk	Low risk	Low risk	Low risk	Serious risk
Thuy et al. [31]	Serious risk	Low risk	Low risk	Low risk	Low risk	Low risk	Low risk	Serious risk

^*∗*^NA: nonapplicable.

**Table 4:** Efficacy of DAA regimens on HCV genotype 5 and 6 patients.

Regimen^*∗*^	Study	Duration (weeks)	Total patients	SVR12 rate (%)	Pooled SVR12 rate (95% CI)^*∗∗*^
*GENOTYPE 5*					
SOF + PR	Lawitz et al. [29]	12	1	100	NA
SOF/VEL	Feld et al. [23]	12	35	97.1	NA
SOF/LDV	Abergel et al. [20]	12	41	95.1	NA
SOF/VEL/VOX	Jacobson et al. [26]	8	18	94.4	NA
*GENOTYPE 6*					
SOF + RBV	Lai et al. [28]	12	3	100	NA
	Lai et al. [28]	16	4	100	NA
	Lai et al. [28]	24	4	100	NA
	Wei et al. [32]	24	4	100	
SOF + PR	Wei et al. [32]	12	32	96.9	**99.6% (92.2%, 100%)**
	Lawitz et al. [29]	12	6	100	
	Kowdley et al. [27]	24	5	100	
SOF/VEL	Everson et al. [22]	12	5	100	NA
	Feld et al. [23]	12	41	100	
	Jacobson et al. [26]	12	9	100	
	Curry et al. [21]	24	1	100	
SOF/VEL 25 mg^*∗∗∗*^	Everson et al. [22]	12	4	100	NA
SOF/LDV	Nguyen et al. [30]	8	20	95	NA
	Nguyen et al. [30]	12	40	95	**99.2% (96.5%, 100%)**
	Gane et al. [24]	12	25	96	
	Thuy et al. [31]	12	86	100	
	Thuy et al. [31]	24	20	100	NA
SOF/LDV + RBV	Thuy et al. [31]	12	39	100	NA
	Thuy et al. [31]	24	30	100	NA
SOF/VEL/VOX	Jacobson et al. [26]	8	30	100	NA
	Gane et al. [25]	8	1	100	
	Gane et al. [25]	12	2	100	NA

SOF, sofosbuvir; VEL, velpatasvir; LDV, ledipasvir; VOX, voxilaprevir; PR, PegIFN + ribavirin; RBV, ribavirin; SVR12, sustained virological response rates at 12^th^ week after treatment; NA, nonapplicable. ^*∗*^Standard dose of each drug was as follows: SOF, 400 mg per day; VEL, 100 mg per day; LDV, 90 mg per day; VOX, 100 mg per day; PegIFN 180 *μ*g per week; RBV 1000–1200 mg per day. ^*∗∗*^The confidence intervals were estimated with the exact method, as recommended by Nyaga et al. [19] who developed the statistical program used for pooling in this study. ^*∗∗∗*^Standard dose of VEL is 100 mg per day. In this study (Everson 2005), a VEL dose of 25 mg per day was experimented.

**Table 5:** Safety of DAA regimens on HCV genotype 5 and 6 patients.

Regimen^*∗*^	Study	Duration (weeks)	Total patients	AE rate (%)	SAE rate (%)	Pooled SAE rate (95% CI)^*∗∗*^
*GENOTYPE 5*						
SOF/LDV	Abergel et al. [20]	12	41	80	2.4	NA
*GENOTYPE 6*						
SOF + RBV	Lai et al. [28]	12	3	—	0	NA
	Lai et al. [28]	16	4	—	0	NA
	Lai et al. [28]	24	4	—	0	NA
SOF/VEL	Curry et al. [21]	24	1	—	0	NA
SOF/LDV	Nguyen et al. [30]	8	20	10	0	NA
	Nguyen et al. [30]	12	40	8	5	**1.7% (0%, 8.2%)**
	Gane et al. [24]	12	25	85	4	
	Thuy et al. [31]	12	86	—	0	
	Thuy et al. [31]	24	20	—	0	NA
SOF/LDV + RBV	Thuy et al. [31]	12	39	—	0	NA
	Thuy et al. [31]	24	30	—	0	NA
SOF/VEL/VOX	Gane et al. [25]	8	1	—	0	NA
	Gane et al. [25]	12	2	—	0	NA

SOF, sofosbuvir; VEL, velpatasvir; LDV, ledipasvir; VOX, voxilaprevir; PR, PegIFN + ribavirin; RBV, ribavirin; AE, adverse event; SAE, serious adverse event; NA, nonapplicable. ^*∗*^Standard dose of each drug was as follows: SOF, 400 mg per day; VEL, 100 mg per day; LDV, 90 mg per day; VOX, 100 mg per day; PegIFN 180 *μ*g per week; RBV 1000–1200 mg per day. ^*∗∗*^The confidence intervals were computed with the exact method, as recommended by Nyaga et al. [19] who developed the statistical program used for pooling in this study.

## Data Availability

The data of search strategy, inclusion and exclusion criteria, data extraction form, and RoB assessment for individual studies, which were used to support the findings of this study, are included within the Appendices.

## Supplementary Materials

Supplementary Materials Appendix 1: search strategy, Appendix 2: inclusion and exclusion criteria, Appendix 3: data extraction form, Appendix 4: additional tables, Appendix 5: additional figures, Appendix 6: PRISMA checklist.
