# Supplementary material for: Systematic Review with Meta-Analysis: Efficacy and Safety of Direct-Acting Antivirals for Chronic Hepatitis C Genotypes 5 and 6
Source: Biomed Res Int. 2019 Nov 11;2019:2301291. doi: 10.1155/2019/2301291 (PMC6877942; doi:10.1155/2019/2301291)
Supplement: Supplementary Materials — Appendix 1: search strategy, Appendix 2: inclusion and exclusion criteria, Appendix 3: data extraction form, Appendix 4: additional tables, Appendix 5: additional figures, Appendix 6: PRISMA checklist. [file 2301291.f1.pdf]

APPENDIX 1. Search strategy

A) Medline (Update: 4th Dec 2018)

**Search**	**Query**	**Items found**
**30**	**Search (#9 AND #14 AND #28 AND #29)**	**359**
**29**	**Search trial**	**1,291,577**
**28**	**Search (#18 OR #21 OR #24 OR #27)**	**2,612**
27	Search (#25 OR #26)	185
26	Search epclusa	31
25	Search velpatasvir	183
24	Search (#22 OR #23)	943
23	Search daklinza	449
22	Search daclatasvir	943
21	Search (#19 OR #20)	872
20	Search harvoni	180
19	Search ledipasvir	864
18	Search (#16 OR #17)	2,191
17	Search solvadi	1
16	Search sofosbuvir	2,190
15	Search (#13 OR #14)	448,067
**14**	**Search genotype**	**448,067**
13	Search (#10 OR #11 OR #12)	379,007
12	Search "genotype 6"	324
11	Search "genotype 5"	295
10	Search "Genotype"[Mesh]	378,825
**9**	**Search (#7 OR #8)**	**84,542**
8	Search HCV	53,817
7	Search (#5 OR #6)	79,089
6	Search "hepatitis C virus"	50,946
5	Search (#3 OR #4)	67,330
4	Search "Hepatitis C, Chronic"[Mesh]	22,384
3	Search (#1 OR #2)	67,330
2	Search “Hepatitis C"[Mesh]	59,529
1	Search "Hepacivirus"[Mesh]	30,654

B) Scopus (Update: 4th Dec 2018)

Search	Query	Items found
**19**	**#3 AND #16 AND #17 AND #18**	**1,051**
**18**	**TITLE-ABS-KEY (trial)**	**2,152,687**
**17**	**TITLE-ABS-KEY (genotype)**	**484,550**
**16**	**#6 OR #9 OR #12 OR #15**	**4,574**
15	#13 OR #14	482
14	TITLE-ABS-KEY (epclusa)	65
13	TITLE-ABS-KEY (velpatasvir)	481
12	#10 OR #11	2,099
11	TITLE-ABS-KEY (daklinza)	115
10	TITLE-ABS-KEY (daclatasvir)	2,096
9	#7 OR #8	2,086
8	TITLE-ABS-KEY (harvoni)	248
7	TITLE-ABS-KEY (ledipasvir)	2,076
6	#4 OR #5	3,971
5	TITLE-ABS-KEY (solvadi)	10
4	TITLE-ABS-KEY (sofosbuvir)	3,970
**3**	**#1 OR #2**	84,720
2	TITLE-ABS-KEY (hcv)	60,849
1	TITLE-ABS-KEY ("hepatitis C virus")	**73,114**

C) The Cochrane Central Register of Controlled Trials (CENTRAL) (Update: 4th Dec 2018)

Search	Query	Items found
**28**	**#7 and #10 and #24 and #27**	**46**
**27**	**#25 or #26**	**530,457**
26	(clinical trial):ti,ab,kw	530,398
25	MeSH descriptor: [Clinical Trial] explode all trees	147
**24**	**#14 or #17 or #20 or #23**	**967**
23	#21 or #22	119
22	(epclusa):ti,ab,kw	8
21	(velpatasvir):ti,ab,kw	119
20	#18 or #19	323
19	(daklinza):ti,ab,kw	5
18	(daclatasvir):ti,ab,kw	323
17	#15 or #16	353
16	(harvoni):ti,ab,kw	23
15	(ledipasvir):ti,ab,kw	351
14	#11 or #12	802
13	(solvadi):ti,ab,kw	-
12	(sofosbuvir):ti,ab,kw	802
11	MeSH descriptor: [Sofosbuvir] explode all trees	153
**10**	**#8 or #9**	**7,408**
9	(genotype 6):ti,ab,kw	5,005
8	(genotype 5):ti,ab,kw	5,456
**7**	**#2 or #4**	**3,886**
6	#3 or #4	2,088
5	#1 or #2	2,999
4	MeSH descriptor: [Hepatitis Viruses] explode all trees	2,088
3	MeSH descriptor: [Hepacivirus] explode all trees	1,233
2	MeSH descriptor: [Hepatitis C] explode all trees	2,999
1	MeSH descriptor: [Hepatitis C, Chronic] explode all trees	1,792

D) The China National Knowledge Infrastructure (CNKI) (Update: 4th Dec 2018)

Search	Query	Items found
1	(SU='hepatitis C' or TI = 'hepatitis C' or KY = 'hepaptis C') and (SU='genotype' or TI = 'genotype' or KY = 'genotype')	396

APPENDIX 2. Inclusion and exclusion criteria

	**Inclusion criteria**	**Exclusion criteria**
**Design**	Clinical trials	Not clinical trials
**Participants**	Included adult participants with **HCV genotype 5 or 6;**<br>Treatment-naïve or treatment –experienced;<br>With or without cirrhosis.	Participants were co-infected with other viruses (e.g. HIV, HBV) or had advanced diseases (e.g. liver or kidney transplantation, hemodialysis).
**Intervention**	Treatment regimens contained at least a **second-generation DAA***	Treatment regimens did not contain any **second-generation DAA**
**Comparator**	Any treatment regimen/ No intervention/ Placebo	
**Outcome**	**SVR12**<br>(Sustained virological response at week 12th after treatment)	Outcomes were not measured by **SVR12**
**Data report**	Reported **SVR12 rate** for HCV genotype 5 and/or 6 participants	Did not report **SVR12 rate**
**Language**	Reported in **English** language	Reported in **non-English** language

* *The second-generation DAAs currently recommended for the treatment of HCV genotypes 5 and 6 include* ***sofosbuvir, ledipasvir, daclatasvir, velpatasvir****. Their trade names are* ***solvadi, harvoni, daklinza, epclusa****, respectively.*

APPENDIX 3. Data extraction form

DATA EXTRACTION FORM

Systematic Review with Meta-analysis: Efficacy and Safety of Direct-Acting Antivirals for Chronic Hepatitis C Genotypes 5 and 6

Data extractor	☐ Due Ong The<br>☐ Anne Julienne M. Genuino
Completion date	/ /

1. **General information**

Study ID (Endnote record)	
First author	
Email address	
Journal	
Publication year	
Funding source	
Study title	

2. **Study design**

Country		
Trial design	☐ Randomized<br>☐ Comparative arms<br>☐ Blind	☐ Non-randomized<br>☐ Single arm<br>☐ Open label

3. **Patient characteristics**

	Genotype 5	Genotype 6
Number of patients		
Age, mean (SD)		
Male, n (%)		
Race, n (%)	White =<br>Black =<br>Asian =<br>Other =	White =<br>Black =<br>Asian =<br>Other =
BMI, mean (SD)		
IL28B genotype, n (%)	CC =<br>CT =<br>TT =	CC =<br>CT =<br>TT =
Prior HCV treatment, n (%)	Treatment-naïve =<br>Non-response =<br>Relapse =	Treatment-naïve =<br>Non-response =<br>Relapse =
Cirrhosis presence, n (%)		
HCV RNA log_{10} IU/mL, mean (SD)		
HCV RNA ≥ 800,000 IU/mL, n (%)		

4. **Treatment characteristic**

	Genotype 5	Genotype 6

**Intervention**		
Regimen (Dose)	☐ SOF/LDV<br>Dose:<br>☐ SOF/VEL<br>Dose:<br>☐ SOF + DCV<br>Dose:<br>☐ SOF + PR<br>Dose:<br>☐ Other:<br>Dose:	☐ SOF/LDV<br>Dose:<br>☐ SOF/VEL<br>Dose:<br>☐ SOF + DCV<br>Dose:<br>☐ SOF + PR<br>Dose:<br>☐ Other:<br>Dose:
Duration		
**Comparator**		
	☐ No comparator	☐ No comparator
Regimen (Dose)	☐ SOF/LDV<br>Dose:<br>☐ SOF/VEL<br>Dose:<br>☐ SOF + DCV<br>Dose:<br>☐ SOF + PR<br>Dose:<br>☐ Other:<br>Dose:	☐ SOF/LDV<br>Dose:<br>☐ SOF/VEL<br>Dose:<br>☐ SOF + DCV<br>Dose:<br>☐ SOF + PR<br>Dose:<br>☐ Other:<br>Dose:
Duration		

5. Outcome reporting

	Genotype 5	Genotype 6
Total number of studied patient	n =	n =
**Efficacy**		
Number of patients achieve SVR12	n =	n =
**Safety**		
Number of patients having SAE	n =	n =
Number of patients having any AE	n =	n =

**Notes:**<br>-<br>-<br>-

APPENDIX 4. Additional tables

Appendix table 1. Efficacy of DAA regimens on HCV genotype 5 and 6 patients, by groups of DAA regimens

Group	Regimen*	Study	Duration *(weeks)*	Total patients	SVR12 rate	Pooled SVR12 rate *(95% CI) ***
**GENOTYPE 5**						
Single DAA regimen	**SOF + PR**	Lawitz (2013) [29]	12	1	100%	NA
Doublet DAA regimen	**SOF/VEL**	Feld (2015) [23]	12	35	97.1%	**96.1% (90.0%, 99.7%)**
	**SOF/LDV**	Abergel (2016) [20]	12	41	95.1%	
Triplet DAA regimen	**SOF/VEL /VOX**	Jacobson (2017) [26]	8	18	94.4%	NA
**GENOTYPE 6**						
Single DAA regimen	**SOF + RBV**	Lai (2016) [28]	12	3	100%	**100% (94.3%, 100%)**
		Wei (2018) [32]	24	4	100%	
	**SOF + PR**	Wei (2018) [32]	12	32	96.9%	
		Lawitz (2013) [29]	12	6	100%	
		Kowdley (2013) [27]	24	5	100%	
Doublet DAA regimen	**SOF/VEL**	Everson (2015) [22]	12	5	100%	**100% (100%, 100%)**
		Feld (2015) [23]	12	41	100%	
		Jacobson (2017) [26]	12	9	100%	
		Curry (2015) [21]	24	1	100%	
	**SOF/VEL** *25mg ****	Everson (2015) [22]	12	4	100%	
	**SOF/LDV**	Nguyen (2017) [30]	12	40	95%	
		Gane (2015) [24]	12	25	96%	
		Thuy (2018) [31]	12	86	100%	
	**SOF/LDV + RBV**	Thuy (2018) [31]	12	39	100%	
Triplet DAA regimen	**SOF/VEL /VOX**	Jacobson (2017) [26]	8	30	100%	**100% (100%, 100%)**
		Gane (2016) [25]	8	1	100%	

SOF, sofosbuvir; VEL, velpatasvir; LDV, ledipasvir; VOX, voxilaprevir; PR, PegIFN + ribavirin; RBV, ribavirin; SVR12, Sustained Virological Response rates at 12th week after treatment; NA, non-applicable.

** Standard dose of each drug was as follows: SOF, 400mg per day; VEL, 100mg per day; LDV, 90mg per day; VOX, 100mg per day; PegIFN 180μg per week; RBV 1000-1200mg per day.*

*** The confidence intervals were estimated with the exact method, as recommended by Nyaga et al. [19] who developed the statistical program used for pooling in this study.*

**** Standard dose of VEL is 100mg per day. In this study (Everson 2005), a VEL dose of 25 mg per day was experimented.*

Appendix table 2. Safety of DAA regimens on HCV genotype 5 and 6 patients, by groups of DAA regimens

Group	Regimen*	Study	Duration *(weeks)*	Total patients	SAE rate	Pooled SAE rate *(95% CI) ** *
**GENOTYPE 5**						
Doublet DAA regimen	**SOF/LDV**	Abergel (2016) [20]	12	41	2.4%	NA
**GENOTYPE 6**						
Single DAA regimen	**SOF + RBV**	Lai (2016) [28]	12	3	0%	NA
Doublet DAA regimen	**SOF/VEL**	Curry (2015) [21]	24	1	0%	**0% (0%, 0%)**
	**SOF/LDV**	Nguyen (2017) [30]	12	40	5%	
		Gane (2015) [24]	12	25	4%	
		Thuy (2018) [31]	12	86	0%	
	**SOF/LDV + RBV**	Thuy (2018) [31]	12	39	0%	
Triplet DAA regimen	**SOF/VEL /VOX**	Gane (2016) [25]	8	1	0%	NA

SOF, sofosbuvir; VEL, velpatasvir; LDV, ledipasvir; VOX, voxilaprevir; PR, PegIFN + ribavirin; RBV, ribavirin; AE, adverse event; SAE, serious adverse event; NA, non-applicable.

** Standard dose of each drug was as follows: SOF, 400mg per day; VEL, 100mg per day; LDV, 90mg per day; VOX, 100mg per day; PegIFN 180µg per week; RBV 1000-1200mg per day.*

*** The confidence intervals were computed with the exact method, as recommended by Nyaga et al. [19] who developed the statistical program used for pooling in this study.*

APPENDIX 5. Additional figures

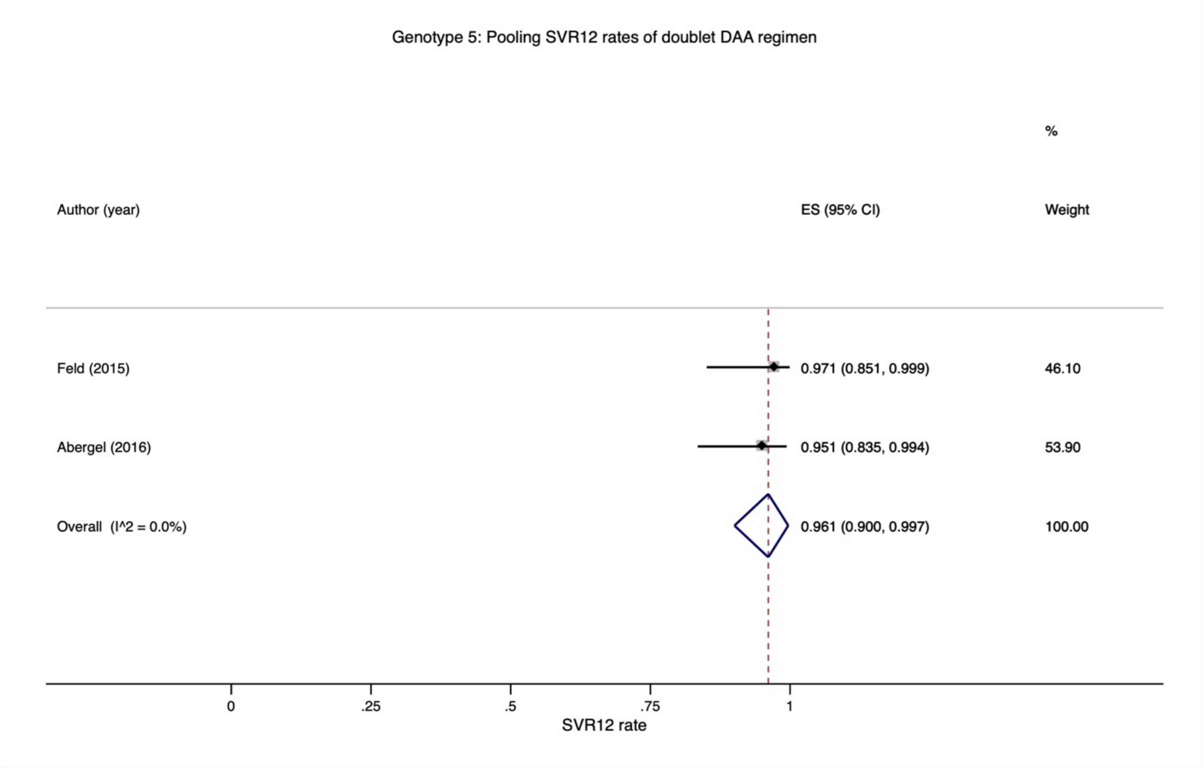

Appendix figure 1. Pooling SVR12 rates of doublet DAA regimens on HCV genotype 5
SVR12, Sustained Virological Response rates at 12 weeks; HCV, Hepatitis C Virus; DAA, Direct Acting Antiviral;

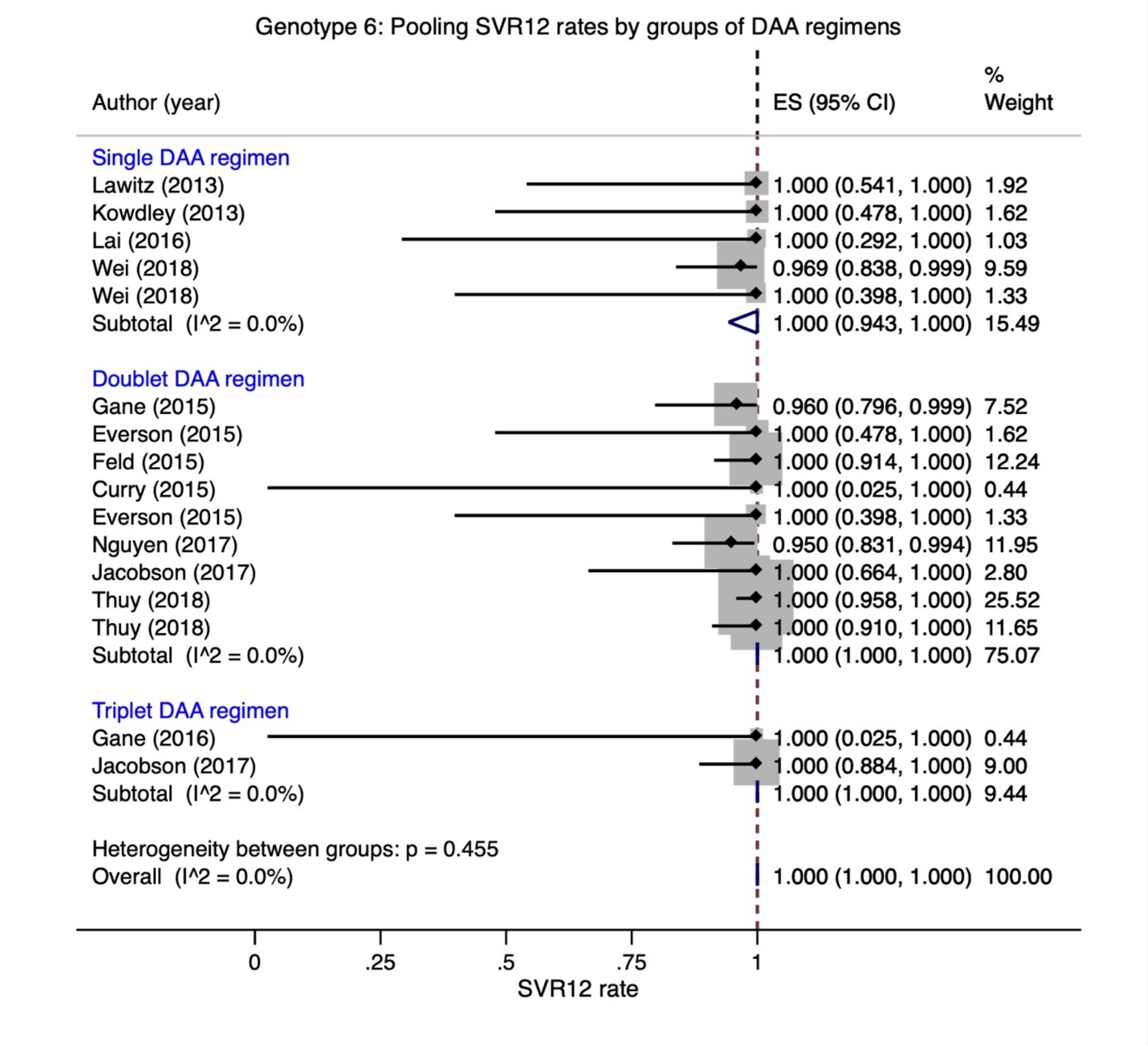

Appendix figure 2. Pooling SVR12 rates by groups of DAA regimens on HCV genotype 6
SVR12, Sustained Virological Response rates at 12 weeks; HCV, Hepatitis C Virus; DAA, Direct Acting Antiviral;

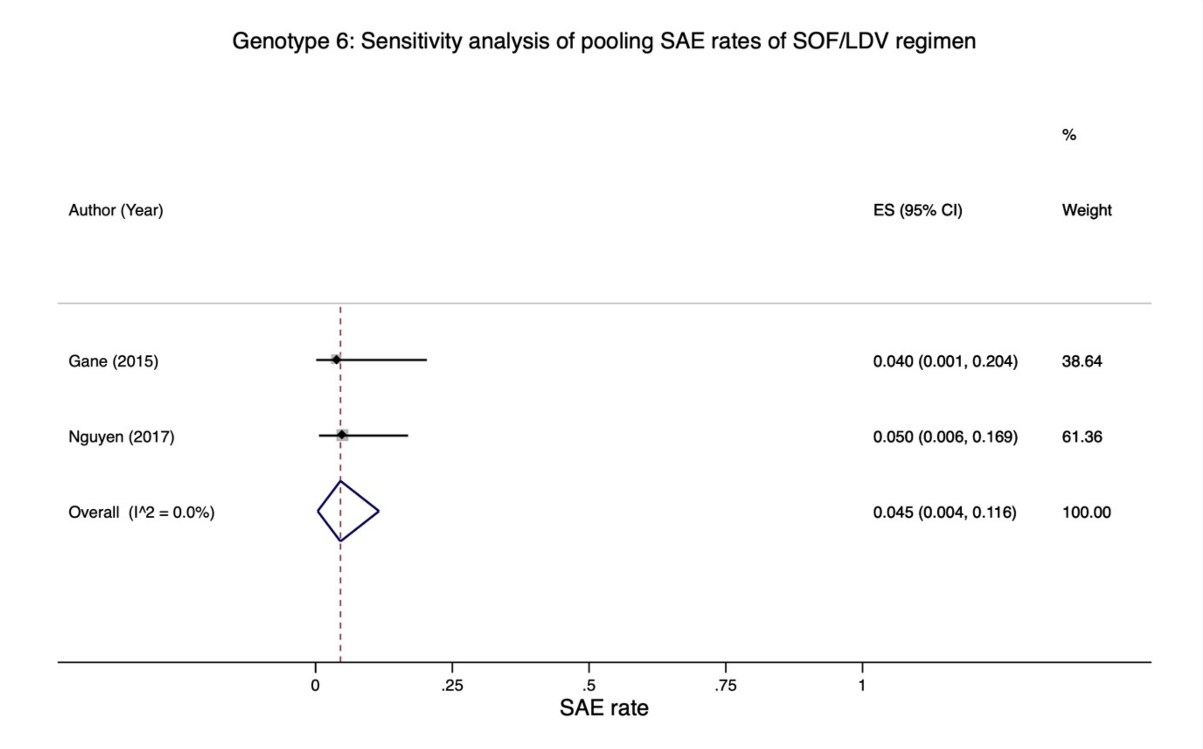

Appendix figure 3. Sensitivity analysis:
Pooling SAE rates of SOFL/LDV regimen on HCV genotype 6
SAE, Serious Adverse Event; HCV, Hepatitis C Virus; SOF, sofosbuvir; LDV, ledipasvir.

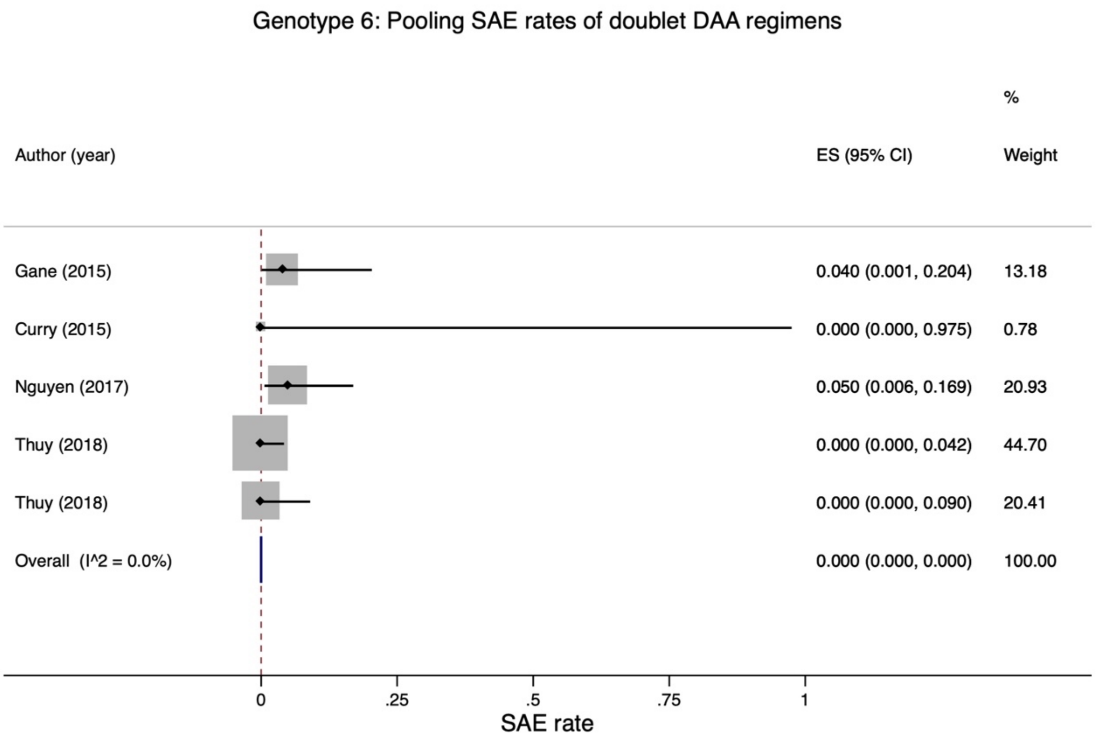

Appendix figure 4. Pooling SAE rates of doublet DAA regimens on HCV genotype 6

SAE, Serious Adverse Event; HCV, Hepatitis C Virus; DAA, Direct Acting Antiviral;

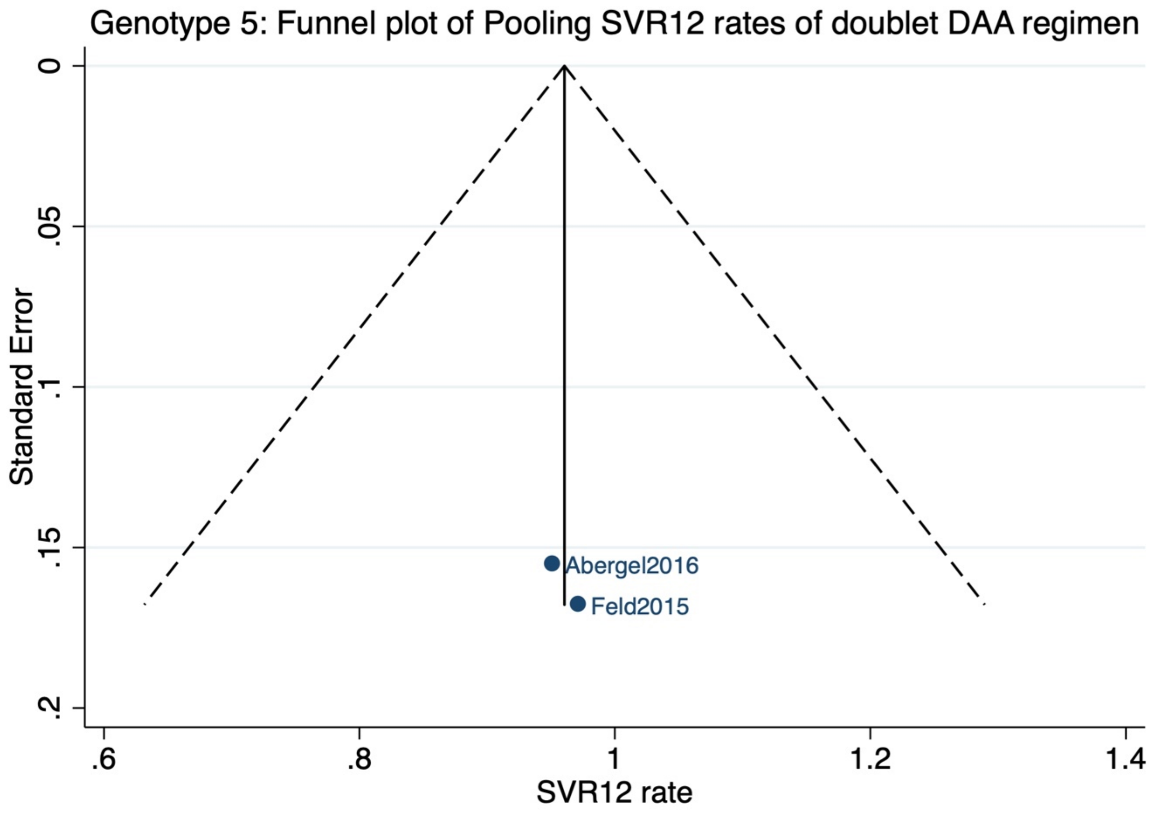

Appendix figure 5. Funnel plot of pooling SVR12 rates of doublet DAA regimens on HCV genotype 5

SVR12, Sustained Virological Response rates at 12 weeks; HCV, Hepatitis C Virus; DAA, Direct Acting Antiviral.

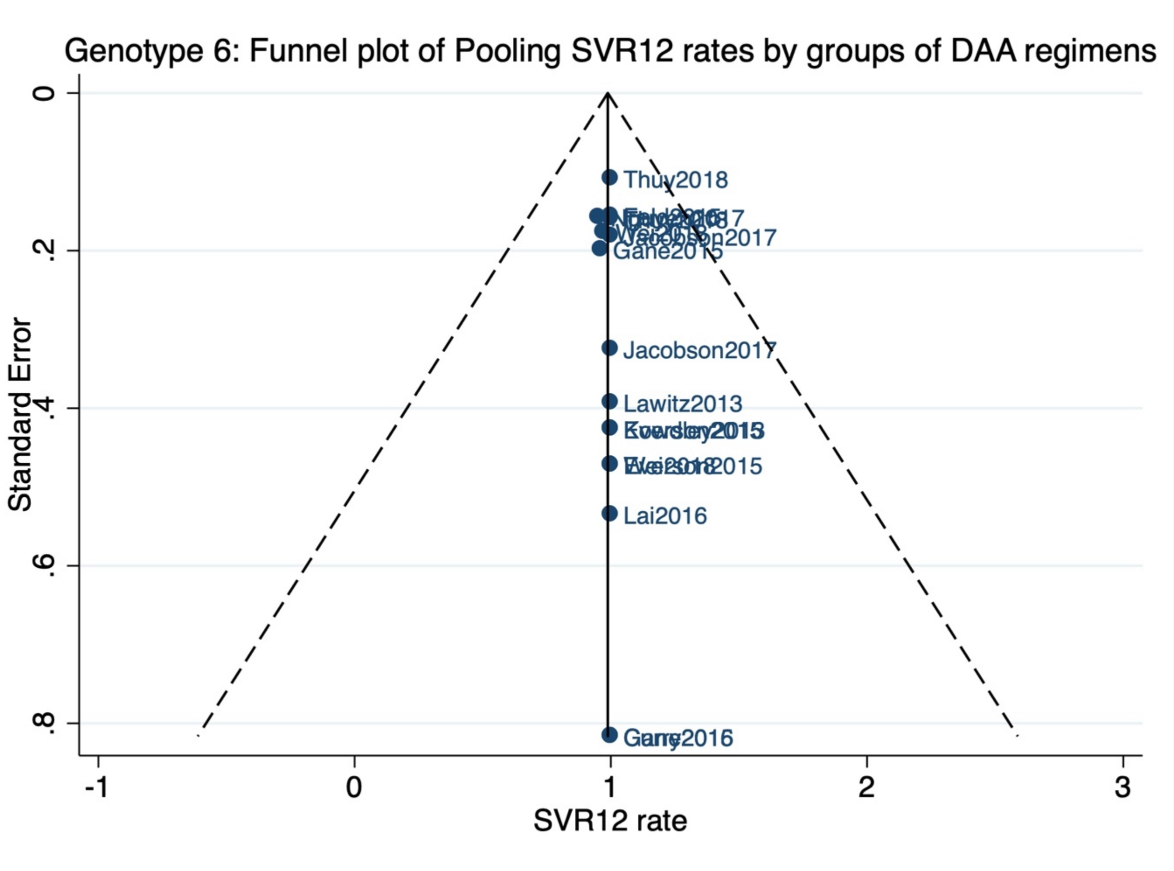

Appendix figure 6. Funnel plot of pooling SVR12 rates by groups of DAA regimens on HCV genotype 6

SVR12, Sustained Virological Response rates at 12 weeks; HCV, Hepatitis C Virus; DAA, Direct Acting Antiviral.

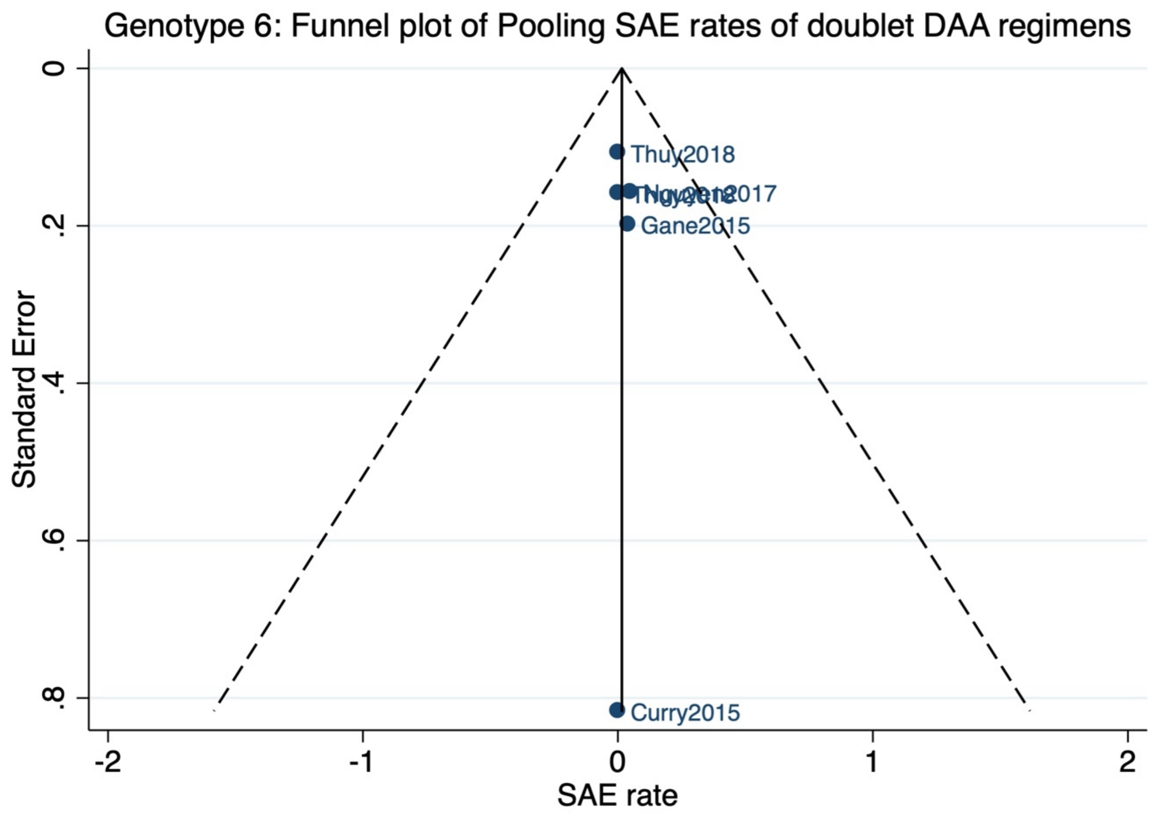

Appendix figure 7. Funnel plot of pooling SAE rates of doublet DAA regimens on HCV genotype 6

SAE, Serious Adverse Event; HCV, Hepatitis C Virus; DAA, Direct Acting Antiviral;

APPENDIX 6. PRISMA checklist

PRISMA 2009 Checklist

Section/topic	#	Checklist item	Reported on page #
**TITLE**			
Title	1	Identify the report as a systematic review, meta-analysis, or both.	1
**ABSTRACT**			
Structured summary	2	Provide a structured summary including, as applicable: background; objectives; data sources; study eligibility criteria, participants, and interventions; study appraisal and synthesis methods; results; limitations; conclusions and implications of key findings; systematic review registration number.	1-2
**INTRODUCTION**			
Rationale	3	Describe the rationale for the review in the context of what is already known.	2-3
Objectives	4	Provide an explicit statement of questions being addressed with reference to participants, interventions, comparisons, outcomes, and study design (PICOS).	3
**METHODS**			
Protocol and registration	5	Indicate if a review protocol exists, if and where it can be accessed (e.g., Web address), and, if available, provide registration information including registration number.	3
Eligibility criteria	6	Specify study characteristics (e.g., PICOS, length of follow-up) and report characteristics (e.g., years considered, language, publication status) used as criteria for eligibility, giving rationale.	3-4, Appendix 2
Information sources	7	Describe all information sources (e.g., databases with dates of coverage, contact with study authors to identify additional studies) in the search and date last searched.	3-4
Search	8	Present full electronic search strategy for at least one database, including any limits used, such that it could be repeated.	3-4, Appendix 1
Study	9	State the process for selecting studies (i.e., screening,	3-4,

Section/topic	#	Checklist item	Reported on page #
selection		eligibility, included in systematic review, and, if applicable, included in the meta-analysis).	Appendix 2
Data collection process	10	Describe method of data extraction from reports (e.g., piloted forms, independently, in duplicate) and any processes for obtaining and confirming data from investigators.	4-5, Appendix 3
Data items	11	List and define all variables for which data were sought (e.g., PICOS, funding sources) and any assumptions and simplifications made.	4-5, Appendix 3
Risk of bias in individual studies	12	Describe methods used for assessing risk of bias of individual studies (including specification of whether this was done at the study or outcome level), and how this information is to be used in any data synthesis.	5
Summary measures	13	State the principal summary measures (e.g., risk ratio, difference in means).	4
Synthesis of results	14	Describe the methods of handling data and combining results of studies, if done, including measures of consistency (e.g., I^2) for each meta-analysis.	5-6
Risk of bias across studies	15	Specify any assessment of risk of bias that may affect the cumulative evidence (e.g., publication bias, selective reporting within studies).	5-6
Additional analyses	16	Describe methods of additional analyses (e.g., sensitivity or subgroup analyses, meta-regression), if done, indicating which were pre-specified.	5-6
**RESULTS**			
Study selection	17	Give numbers of studies screened, assessed for eligibility, and included in the review, with reasons for exclusions at each stage, ideally with a flow diagram.	6, Figure 1
Study characteristics	18	For each study, present characteristics for which data were extracted (e.g., study size, PICOS, follow-up period) and provide the citations.	6-8, Table 1
Risk of bias within studies	19	Present data on risk of bias of each study and, if available, any outcome level assessment (see item 12).	8-9, Table 2A, 2B
Results of	20	For all outcomes considered (benefits or harms), present,	10-15,

Section/topic	#	Checklist item	Reported on page #
individual studies		for each study: (a) simple summary data for each intervention group (b) effect estimates and confidence intervals, ideally with a forest plot.	Table 3-4, Figure 2-3
Synthesis of results	21	Present results of each meta-analysis done, including confidence intervals and measures of consistency.	10-15, Table 3-4, Figure 2-3
Risk of bias across studies	22	Present results of any assessment of risk of bias across studies (see Item 15).	15-16, Figure 4
Additional analysis	23	Give results of additional analyses, if done (e.g., sensitivity or subgroup analyses, meta-regression [see Item 16]).	Appendix 4-5
**DISCUSSION**			
Summary of evidence	24	Summarize the main findings including the strength of evidence for each main outcome; consider their relevance to key groups (e.g., healthcare providers, users, and policy makers).	16-18
Limitations	25	Discuss limitations at study and outcome level (e.g., risk of bias), and at review-level (e.g., incomplete retrieval of identified research, reporting bias).	18-19
Conclusions	26	Provide a general interpretation of the results in the context of other evidence, and implications for future research.	19
**FUNDING**			
Funding	27	Describe sources of funding for the systematic review and other support (e.g., supply of data); role of funders for the systematic review.	19

From: Moher D, Liberati A, Tetzlaff J, Altman DG, The PRISMA Group (2009). Preferred Reporting Items for Systematic Reviews and Meta-Analyses: The PRISMA Statement. PLoS Med 6(6): e1000097. doi:10.1371/journal.pmed1000097

For more information, visit: **www.prisma-statement.org**.
